# Oral metronomic chemotherapy as a feasible preoperative therapy in advanced resectable oral cavity squamous cell carcinomas— a preliminary experience

**DOI:** 10.3332/ecancer.2022.1425

**Published:** 2022-07-07

**Authors:** V P Praveen Kumar Shenoy, Avaronnan Manuprasad, Sajith Babu, Sithara Aravind, Vinin N Narayanan, Sangeetha Nayanar, Satheesan Balasubramanian

**Affiliations:** 1 Department of Clinical Hematology and Medical Oncology, Malabar Cancer Centre, Thalassery, Kannur, Kerala 670103, India; 2 Department of Surgical Oncology, Malabar Cancer Centre, Thalassery, Kannur, Kerala 670103, India; 3 Department of Head and Neck Oncology, Aster MIMS Kannur, Kannur, Kerala, India; 4 Department of Oncopathology, Malabar Cancer Centre, Thalassery, Kannur, Kerala 670103, India; 5 Department of Radiation Oncology, Malabar Cancer Centre, Thalassery, Kannur, Kerala 670103, India

**Keywords:** oral cavity cancer, preoperative therapy, metronomic chemotherapy, waiting times, LMIC

## Abstract

**Introduction:**

Surgery is an important component of multimodality treatment in advanced oral cavity cancers. But in low-middle-income countries like India, with limited centres offering complex head and neck surgeries, prolonged waiting times for surgery is a major problem. An increase in waiting times for treatment has been shown to be a negative prognosticator in head and neck cancer and many patients can develop interim progression making them ineligible for radical treatment. We share our preliminary experience of using oral metronomic chemotherapy as a preoperative treatment in patients expecting delay in surgery.

**Methods:**

This was a retrospective analysis of case records of patients with resectable Stage III and Stage IV (IVA & IVB) oral cavity cancers who had received preoperative oral metronomic chemotherapy (POMT). The POMT schedule consisted of oral Methotrexate 15 mg/m2 weekly, Celecoxib 200 mg twice daily and Erlotinib 100 mg daily. Clinico-radiological assessments were done prior to surgery using standard response assessment criteria.

**Results:**

A total of 68 patients received POMT with a median age of 55 years (range: 34–73 years). Forty-eight (70%) were males, 29 (42%) had carcinoma tongue and majority (*N* = 52, 76%) had Stage IVA cancer. Mean duration of POMT administration was 30.45 days (standard deviation: 8.22). Thirty-seven (54%) patients had partial responses and another 23 (34%) had stable disease. Two (3%) had disease progression on POMT. Fifty-eight (85%) underwent surgery after POMT. Margin positive resection was seen in two patients. Half of the patients who received POMT did not experience any toxicity. Grade 3/4 toxicities were seen in four (6%) patients.

**Conclusions:**

POMT is a feasible strategy worth considering in cases where there are prolonged waiting times to surgery.

## Introduction

Oral cavity cancer is one of the most common incident cancers in India and along with lip cancer, it accounted for 10.3% of all cancers in India as per GLOBOCAN 2020 [1]. About 60% of oral cavity cancers present in advanced stages and may require multimodality treatment including complex surgeries. Prolonged waiting time for surgery is an important hurdle in delivering the optimal treatment all over the world. In a country like India where there are a limited number of centres offering comprehensive cancer treatment, the magnitude of this problem can be manifold [2]. Increase in waiting time for treatment has been shown to be a negative prognosticator in head and neck cancers [3, 4]. It has been reported that within an average time of 4 weeks, up to 30% can have progressive disease (PD) and 16% can have progression as per TNM (Tumour, Node, Metastasis) classification and it may make many patients ineligible for any form of curative treatment [5].

Surgery is the first treatment in the management of the majority of oral cavity cancers. Waiting times for surgery can be managed by instituting some treatment that would prevent tumour progression and maintain it in an operable stage and also ensuring that such treatment in itself does not delay the definitive surgery. Such treatment should be easily deliverable, minimally or totally non-toxic and economical. The recommended induction chemotherapy regimens for head and neck cancer are highly toxic and hence it is not feasible to use them during the waiting period. Metronomic chemotherapy which has emerged as a standard of care in metastatic disease is an attractive option in such situations. Metronomic chemotherapy is the administration of low, minimally toxic doses of chemotherapeutic agents with no prolonged drug-free breaks [6]. This was developed originally to overcome drug resistance by targeting tumour vasculature. Recent studies have shown that metronomic therapy exerts anticancer activity in multiple ways. Metronomic therapy activates immunity against cancer cells by inhibiting T regulatory cells, inducing dendritic cell maturation and increasing lymphocyte proliferation and memory T cells. Metronomic chemotherapy has been shown to induce tumour dormancy and senescence as well [7].

Metronomic chemotherapy has been shown to be a low-cost and non-inferior option compared to single-agent cisplatin in recurrent metastatic and inoperable head and neck cancers [8]. Low rates of serious toxicities coupled with reasonable progression-free survival seen in metastatic settings prompted us to use metronomic scheduling of chemotherapy during waiting times to definitive surgery [9–11]. Here we describe our preliminary experience in utilising preoperative oral metronomic chemotherapy (POMT) during waiting times to surgery.

## Materials and methods

This was a retrospective analysis of case records of patients with resectable Stage III or Stage IV (IVA & IVB) oral cavity cancers who had received POMT. Approval was obtained from the institutional review board (IRB) for this analysis and a waiver of consent was obtained in view of the retrospective nature of the study. IRB Number – 1616/IRB-SRC/13/MCC/13-04-2019/4. We initiated the administration of POMT since the waiting period in our hospital was more than 3 weeks. This was because our centre is a tertiary cancer centre in the public-sector catering to a large number of patients in our state. Patients with Stage III and Stage IV oral cavity squamous cell carcinomas were counselled on the option of POMT during the waiting periods for surgery after the concurrence in multipseciality tumour board. Patients with borderline resectable oral cavity cancers were excluded. Informed consent was obtained prior to the initiation of metronomic therapy after patients were given the earliest possible provisional surgery date. Baseline assessments included complete blood count, renal function tests, liver function tests, random blood sugar, imaging of primary site and CT chest (to exclude lung metastasis). CT scan of head and neck with contrast was the imaging modality for all primary sites except in case of tongue where MRI with contrast was performed. The POMT schedule consisted of oral Methotrexate 15 mg/m^2^ weekly, oral Celecoxib 200 mg twice daily and Erlotinib 100 mg once daily. Lower dose of Erlotinib was used in our study to reduce the incidence of skin related toxicity. Celecoxib was avoided in patients who had a documented history of coronary artery disease. All the patients on POMT were reviewed every 2 weeks to assess for clinical response and toxicities. Methotrexate was stopped a week prior to the provisional surgery date. Other two medications were allowed to continue till surgery. Response was assessed clinically as well as radiologically on the day of admission to hospital for surgery. Clinical response status was recorded using the clinical criteria of the World Health Organization. Standard Response Evaluation Criteria in Solid Tumours (RECIST) criteria were used for radiological response assessment. Toxicity of POMT was graded according to the National Cancer Institute Common Toxicity Criteria Version 4.03. The patients then underwent surgery as warranted by the disease, ensuring wide resection with margins more than 5 mm. The histopathology was assessed, and the patients were given adjuvant therapy (radiation or chemoradiation) as per standard oncological indications.

## Results

### Demographics

A total of 68 patients received POMT and had a median age of 55 years (range: 34–73 years). Forty-eight (70%) were males while 20 (30%) were females. More than a third had carcinoma tongue (*N* = 29, 42%) and the majority (*N* = 52, 76%) had Stage IVA cancer as per the 8th edition of American Joint Committee on Cancer-TNM staging system. Other baseline details are given in Table 1.

### Efficacy and outcomes

Three drug regimens were given to all patients except two patients who had a history of coronary artery disease where Celecoxib was omitted. The mean duration of POMT administration was 30.45 days (standard deviation: 8.22).

More than half (54%) of our patients had partial responses (PRs) to POMT. Response details are given in Table 2. Clinical benefit rate (PR + stable disease (SD)) was seen in 60 (88%) patients. Fifty-eight (85%) underwent surgery after POMT and this included one patient who progressed on POMT but was operable. Eight patients defaulted prior to surgery and one patient opted for the continuation of metronomic chemotherapy. Margin-positive resection was seen in two patients. One (1.4%) patient had a pathological complete response. Adjuvant radiation was given to 54 patients. Concurrent chemotherapy was administered along with adjuvant radiation in 23 patients. Forty-nine patients completed planned adjuvant treatment in the form of radiation and/or chemotherapy.

### Toxicity

Half of the patients (*N* = 36, 53%) who received POMT did not experience any toxicity. Major toxicities observed included skin rash in 19 (28%) patients, mucositis in 12 (18%) while one patient had both mucositis and skin rash. Grade 3/4 toxicities were seen in 4 (6%) patients. Three patients had grade 3 mucositis while one had grade 3 skin rash. Other toxicities observed were grade 2 gastritis in one patient, grade 2 nausea and vomiting in one patient, grade 1 transaminitis in one, and one patient developed grade 2 increase in serum creatinine.

Due to toxicities observed, dose reduction had to be done in three patients while single or multiple doses of one or more drugs had to be skipped in seven patients. In one patient, POMT had to be discontinued completely due to toxicity in a patient who developed grade 3 mucositis and grade 2 derangement in renal function. Methotrexate dose had to be reduced in three patients. Two patients had an interruption of POMT by more than a week.

## Discussion

Our study suggests that triple metronomic chemotherapy is a feasible preoperative treatment strategy for patients with operable advanced oral cavity cancers awaiting surgery, especially in a resource-limited setting. The combination of methotrexate, celecoxib and erlotinib could produce a clinical benefit rate close to 90%. Most of the patients in our study had Stage IV cancer and the most common site was the tongue followed by buccal mucosa and alveolus. Importantly most of the patients could proceed with definitive surgery and only 3% had progression while on metronomic chemotherapy. POMT was well tolerated and only one patient required complete discontinuation of POMT due to significant toxicity.

There are only very few studies that utilised metronomic chemotherapy in the preoperative setting among head and neck cancer patients. In a retrospective matched-pair analysis by Pai *et al* [11], a combination of methotrexate and celecoxib was used preoperatively in 32 patients with advanced operable head and neck cancers. The response rate was about 75% and the median duration of therapy was 5 weeks. The longer duration of chemotherapy might be the cause for higher response rates observed in their study compared to our study. In a phase 2 study, erlotinib and celecoxib were used for a treatment period of 21 days in patients with operable oral cancers and had a response rate of 60% which is comparable to our study [12]. The clinical benefit rate was also similar to our study (85% versus 88%). Preoperative scheduling of metronomic chemotherapy has been tried in advanced borderline operable head and neck cancers and responses have been observed in the range of 27% [13]. In this study by Sultania *et al* [13], among 60 patients who received preoperative metronomic therapy, 43% were resectable at the end of 8 weeks of therapy. A multicentre prospective study published recently evaluated the role of two-drug metronomic therapy in multiple settings including patients expecting delays in surgery of more than 2 months [14]. In the study, 11% of the patients had progressive disease at the end of 8 weeks, whereas, in our study, with a mean duration of treatment of about 4 weeks, only 3% had interim progression. This shows the need of identifying the optimum duration of POMT to achieve the best result.

The response rates with metronomic chemotherapy are significantly lesser compared to the well-studied induction regimens like TPF (Docetaxel, Cisplatin and 5-Fluorouracil). But as the clinical benefit rate was high, most of the patients receiving POMT also could proceed with curative surgery. In the study by Zhong *et al* [15], where two cycles of induction chemotherapy were used, there was no overall survival benefit despite a response rate of 80%. Also, it was a regimen requiring hospitalisation and various supportive measures including prophylactic antibiotics. Almost one-third of the patients had haematological toxicity and about 7% had grade 3/4 toxicity [14]. Though we used three drugs unlike prior studies in the preoperative setting, it was well tolerated, and Grade 3/4 toxicities were observed in less than 1% of patients. Only one patient required a complete stoppage of POMT in view of toxicities. In the study by Pai *et al* [11], no major toxicities were reported with POMT. In the Phase 2 study by Nair *et al* [12], grade 2/3 rash was observed with the combination of Erlotinib with celecoxib [12].

To the best of authors’ knowledge, this is the first study reporting the utility of triple metronomic therapy as a bridge prior to definitive surgery. Our preliminary experience shows that triple metronomic therapy is a low-cost, minimally toxic and effective preoperative treatment strategy useful in a resource-limited setting with prolonged waiting times for surgery. This strategy also gains importance in the current COVID pandemic situation where successive waves have placed healthcare under unprecedented stresses resulting in the delay of many routine procedures especially elective cancer surgeries. Though there are limitations being a single-centre retrospective study with short follow-up, our experience offers a promising option to address the problems related to prolonged waiting times in head and neck cancer surgeries.

## Conclusions

Metronomic chemotherapy using the combination of methotrexate, erlotinib and celecoxib is a low-cost, safe and effective preoperative therapy in patients with advanced operable oral cavity cancers in a resource-constrained setting with prolonged waiting times for surgery. Larger randomised trials are required to determine the exact benefit and duration of metronomic therapy in the preoperative setting.

## Funding

There were no external sources of funding for this project.

## Conflicts of interest

The authors declare that they have no conflicts of interest.

## Figures and Tables

**Table 1.** Baseline characteristics.

Baseline characteristics (*N* = 68)	Numbers (%)
Median age	55 years (range: 34–73)
Gender – male: female	48:20 (70:30)
Site Tongue Buccal mucosa Alveolus Lip Gingiva Floor of mouth	29 (43) 18 (26) 16 (23) 1 (2) 1 (2) 3 (4)
Stage III IV A IV B	12 (18) 52 (76) 4 (6)
Histology PD SCC MD SCC WD SCC	5 (7) 47 (69) 16 (24)

SCC, Squamous cell carcinoma; PD, Poorly differentiated; MD, Moderately differentiated; WD, Well differentiated

**Table 2.** Response to POMT.

Responses	Clinico-radiological
PR	37 (54%)
SD	23 (34%)
PD	2 (3%)
Missing data/defaulted	6 (9%)

POMT, Preoperative metronomic chemotherapy
